# Uveitis prior to clinical presentation of Multiple Sclerosis (MS) is associated with better MS prognosis

**DOI:** 10.1371/journal.pone.0264918

**Published:** 2022-06-29

**Authors:** Eyal Raskin, Anat Achiron, Ofira Zloto, Ron Neuman, Vicktoria Vishnevskia-Dai

**Affiliations:** 1 Ophthalmology, Barzilai Medical Center, Askelon, Israel; 2 Faculty of Health Science, Ben Gurion University of the Negev, Beersheba, Israel; 3 Multiple Sclerosis Institute, Sheba Medical Center, Tel Hashomer, Israel; 4 Sackler Faculty of Medicine, Tel Aviv University, Tel Aviv, Israel; 5 Ocular Oncology and Inflammatory Eye Disease Service, Goldshleger Eye Institute, Tel Hashomer, Israel; 6 Maccabi Healthcare Service, Tel Aviv, Israel; IRCCS San Raffaele Scientific Research Institute, ITALY

## Abstract

**Objective:**

An association between uveitis and multiple sclerosis (MS) is well-established, but the actual nature of that association remains poorly understood. We sought to determine the association between the presence of a uveitis diagnosis prior to an MS diagnosis compared to no pre-existing uveitis diagnosis in MS patients.

**Methods:**

Patients in whom the presentation of uveitis preceded the presentation of MS (**study group**) and patients with MS and no uveitis (**control group**) were randomly selected at a ratio of 1:3 from the Sheba Multiple Sclerosis Center computerized database.

**Results:**

Eleven patients presented with uveitis prior to MS diagnosis (study group), and 31 randomly selected patients had MS without uveitis (control group). Only one patient in the study group deteriorated to EDSS 3 during the follow-up period, compared to 15 patients in the control group (9.1% vs 48.4%, P = 0.049). None of the patients in the study group reached EDSS 6 during the 10 years of follow-up compared to 6 (19.4%) patients in the control group (P = 0.194).

**Conclusions:**

MS patients who presented with uveitis that preceded their neurological symptoms of MS demonstrated a clinically significant better neurological prognosis, than our MS patients with no uveitis.

## Introduction

Uveitis is a sight-threatening intraocular inflammation that affects both the uveal tract and adjacent structures. Uveitis is classified anatomically into anterior, intermediate, posterior, and pan-uveitis forms based on the part of the eye that is primarily affected. Uveitis may be associated with a variety of diseases, including distinct ocular syndromes, immunologically mediated systemic diseases, infections, and masquerading syndromes, such as malignancies [1].

Multiple sclerosis (MS) is an immune-mediated chronic inflammatory disease of the central nervous system (CNS). It usually affects young adults between 20 and 40 years of age and is the leading non-traumatic cause of nervous system disability in this age group. Clinically, MS is characterized by repeated episodes (relapses) of nervous system symptoms and signs, followed by remission (relapsing–remitting MS [RRMS], 80–90% of cases) or by continuous progression of symptoms or signs (primary progressive MS [PPMS], 10–20% of cases) [2]. The Expanded Disability Status Scale (EDSS) is used to describe disease progression in patients with MS. It consists of an ordinal rating system ranging from 0 (normal neurological status) to 10 (death due to MS). EDSS 3 and EDSS 6 represent minor and major neurological disability attributed to MS, respectively.

Early determination of clinical, magnetic resonance imaging (MRI), and/or biological markers that are prognostic for long-term outcomes would be valuable in order to enhance management strategies tailored to the needs of individual patients. Currently, no baseline or short-term clinical, laboratory or MRI measures have proven to be consistent in providing such essential information [3].

The association between uveitis and MS has been known for several decades. The frequency of uveitis in MS patients ranges from 0.4 to 26.9%, and the prevalence of MS in patients with uveitis is 1%-14% [4, 7]. The subtype of uveitis that is most commonly associated with MS is “intermediate” uveitis. Although this association is well-documented, the pathophysiology that explain the association remains poorly understood [5]. Torre et al have even suggested a possible common genetic pattern of expression [6]. Additional explanation to the association is a common genetic pattern of expression. MS and uveitis are both associated with expression of the HLA-DRB1*15:01 haplotype, a subtype of HLA-DR2.

In a retrospective cross sectional study, Shugaiv et al suggested that uveitis presented prior or after MS diagnosis might be used as a good prognostic factor for MS [7]. Lynn et al reported that patients who have known MS carry an approximately 1% risk of developing clinical intraocular inflammatory disease [8]. We sought to determine the association between the presence of a uveitis diagnosis prior to an MS diagnosis and no pre-existing uveitis diagnosis in MS patients. We hypothesized that the link might involve disease progression in favor of the former.

## Methods

### Study design

We conducted a historical cohort study in order to evaluate the progression of MS in patients with and without a uveitis diagnosis prior to their MS diagnosis.

### Setting

The Goldschlager Eye Institute and the Multiple Sclerosis Center of Sheba Medical Center are university-affiliated tertiary care centers. The study was approved by the local IRB.

### Participants

All patients aged 18 years and above with MS-associated uveitis that had presented before their MS symptoms and who were treated at the Sheba Medical Center between 1963 and 2012 were suitable for study entry (**the study group**). The patients who had been diagnosed with MS only and no uveitis (**the comparison group**) were randomly selected at the ratio of 1:3 from the Sheba Multiple Sclerosis Center computerized database. Patients were included in the study if they had undergone comprehensive neurological and ophthalmic examinations at the time of diagnosis, and if their Expanded Disability Status Scale (EDSS) scores were less than EDSS 3 at presentation. Patients who had an EDSS score of 3 or higher at the first presentation were excluded from the study (since this variable was the outcome measure). All of the study participants were followed for up to 10 years.

### Variables

Patient characteristics at baseline that were retrieved from their medical charts included demographics (sex, age at diagnosis), concomitant general illness, and concomitant ophthalmic diseases/conditions (glaucoma, cataract, and others). Outcome measurements were evaluated with the EDSS. The first episode during which the patient reached EDSS 3 (moderate neurological disability attributed to MS) and EDSS 6 (major neurological disability attributed to MS) were recorded.

### Sample size

A total of 40 patients were needed to reach a power of 80% and significance of 5% based on previous publications and our long term clinical impression.

### Quantitative variables

Anterior uveitis severity was assessed by cell count and flare evaluation in accordance with the Standardization of Uveitis Nomenclature (SUN) classification [9]. Intermediate uveitis severity was assessed by vitreous cell count and haze evaluation, which was also in accordance with the SUN classification. The presence of posterior uveitis vasculitis, retinitis, and choroiditis were recorded as either present or absent as recorded in the patients chats based on the clinical examination and in additionally on imaging studies i.e. Optical Coherence Tomography (OCT) and fluorescein angiogram (FA) see Table 1 for detailed ophthalmic presentation at diagnosis (Dx) and at study entry (Ex).

**Table 1:** Treatment array of the patients in the study and control groups.

	MS+UV (n = 11)		MS (n = 31)		p
Prednisone treatment Ex*	1	(9.1%)	0	(0.0%)	0.262
IVIG treatment Ex*	1	(9.1%)	3	(9.7%)	>0.999
IFN treatment Ex*	4	(36.4%)	8	(25.8%)	0.699
Copaxone treatment Ex*	0	(0.0%)	3	(9.7%)	0.564
Bblock treatment Ex*	0	(0.0%)	0	(0.0%)	NA
ACEֹI treatment Ex*	1	(9.1%)	0	(0.0%)	0.262
Statins treatment Ex*	1	(9.1%)	0	(0.0%)	0.262
Calcium+D3 treatment Ex*	0	(0.0%)	0	(0.0%)	NA
Antiacid treatment Ex*	0	(0.0%)	0	(0.0%)	NA
Aspirin treatment Ex*	1	(9.1%)	0	(0.0%)	0.262
DM treatment Ex*	0	(0.0%)	0	(0.0%)	NA
Antimetabolites treatment Ex*	2	(18.2%)	0	(0.0%)	0.064
AntiTcells treatment Ex*	1	(9.1%)	0	(0.0%)	0.262
Other neurological treatment Ex*	2	(18.2%)	0	(0.0%)	0.064
Biphosphonate treatment Ex*	1	(9.1%)	0	(0.0%)	0.262
Hormonal treatment Ex*	0	(0.0%)	0	(0.0%)	NA
Tysabri treatment Ex*	0	(0.0%)	3	(9.7%)	0.554
Gilenya treatment Ex*	0	(0.0%)	1	(3.2%)	>0.999

### Statistical analysis

Categorical variables were described by frequency and percentage. Continuous variables were evaluated for normal distribution with a histogram. Normally distributed continuous variables were described by means and standard deviations, and non-normally distributed continuous variables were described as median and interquartile ranges (IQR). Categorical variables at baseline were evaluated by mens of the Chi-square or Fisher’s exact test. Continuous variables were evaluated with the t-test and the Mann Whitney test. The propensity score was calculated as the probability of having uveitis. A logistic regression was applied to create the propensity score based on patient age and gender. Survival analysis evaluated the association between the study group and the first EDSS of 3 or an EDSS of 6 during the follow-up period as assessed by Kaplan–Maier curves, the log rank test, and multivariate Cox regression. The multivariate analysis was regressed twice, once with age and gender as potential confounders and once with the propensity score. Moreover, since the sample size is small due to the rarity of the medical condition, we also analyzed our data by using the bootstrapping method with 1000 repetitions. All statistical analyses were performed with IBM SPSS for Windows version 21. *P*<0.05 was considered statistically significant. All statistical analyses were 2-tailed.

## Results

Fifty-six patients with MS who were ≥18 years of age were identified for inclusion in the study. Fourteen were excluded due to uveitis onset after MS diagnosis, leaving a total of 42 suitable patients. Of them, 31 patients had MS without uveitis (the control group) and 11 patients who were diagnosed with uveitis prior to their MS diagnosis (the study group).

**Fig 1:**
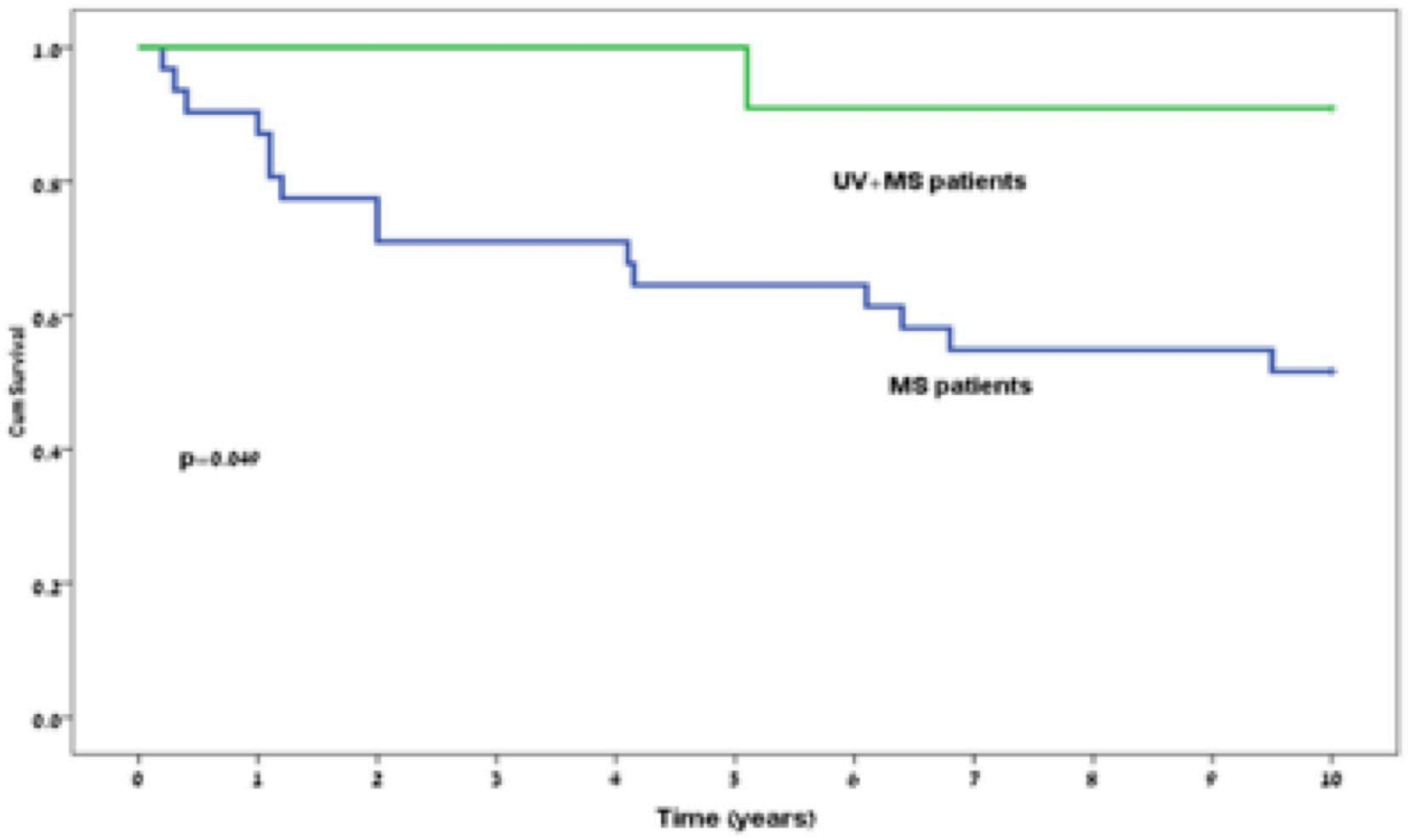
Kaplan-Meier curve of expanded disability status scale 3 incidence during follow-up.

**Fig 2:**
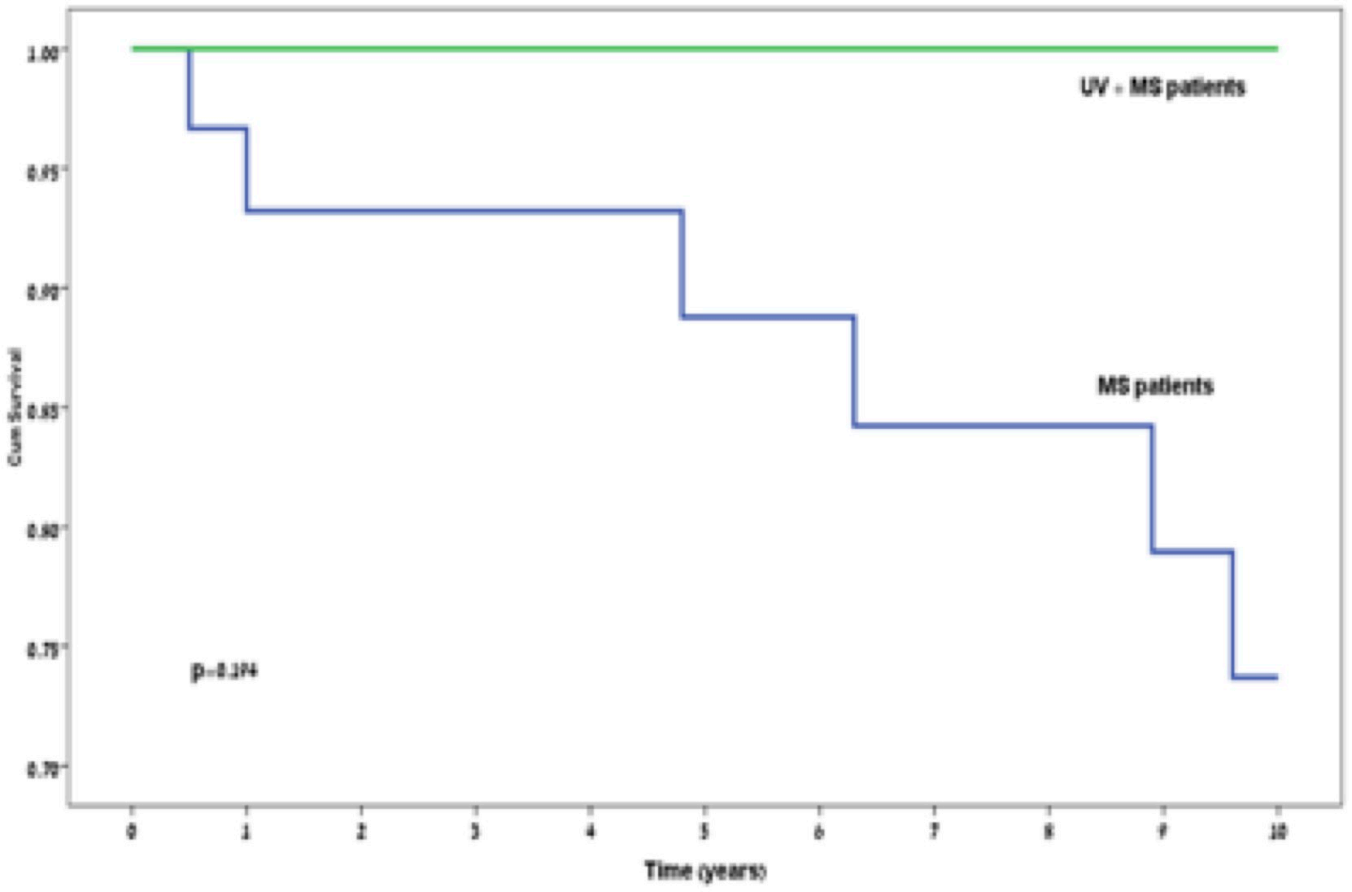
Kaplan-Meier curve of expanded disability status scale 6 incidence during follow-up.

The patients’ characteristics are described in Tables 2 and 3. Those in the control initially presented with more neurological sensory signs compared to the study group (64.5% vs. 27.3%, respectively, *P* = 0.043). There were no other factors that were significantly different between the 2 groups. Only one patient in the study group deteriorated to EDSS 3 during the follow-up period compared to 15 patients in the control group (9.1% vs 48.4%, respectively, *P* = 0.049). None of the patients in the study group reached EDSS 6 during the 10-year follow-up period compared to 6 (19.4%) of the participants in the control group, who reached EDSS 6 (*P* = 0.194). Figs 1 and 2 demonstrate the EDSS 3 and EDSS 6 incidences over time. The median follow-up time was 7.75 years.

**Table 2:** Patient characteristics and neurological status at baseline.

	Patients with Uveitis (N = 11)			Patients without Uveitis (N = 31)	*P*
Age (years), median (IQR)	33.3 (25.20–39.92)		29.41 (24.56–5.21)		0.222
Male, *n*	6 (54.50%)		14 (45.20%)		0.592
Glaucoma, *n*	0 (0%)		0 (0%)		NA
IOP, *n*	0 (0%)		0 (0%)		NA
First neurological signs, *n*	Sensory	3 (27.3%)	20 (64.50%)		**0.043** *
	Sphincter	0 (0%)	4 (12.90%)		0.558
	Motor	3 (30.0%)	12	(38.70%)	0.72
	Cerebellar	1 (9.10%)	2	(6.50%)	>0.999
	Cognitive	0 (0.00%)	0	(0.00%)	NA
	Brainstem	2 (18.20%)	1	(3.20%)	0.163
	Optic neuritis	5 (45.50%)	10	(32.30%)	0.481

IOP = Intraocular pressure; IQR = interquartile range; NA = not applicable

*Significant.

**Table 3:** Clinical features of patients with MS and uveitis.

	MS+UV	UV	p
	(n = 25)	(n = 13)	MS+UV vs. UV
UV diagnosis (in years), medain (iqr)	13.23 (6.09–21.54)	4.94 (2.17–8.55)	**0.044**
Visual acuity (logMAR) Ex*, medain (iqr)	0.20 (0.05–0.40)	0.00 (0.00–0.25)	0.097
Glaucoma Ex*	3 (12.0%)	0 (0.0%)	0.538
DM, n(%)	0 (0.0%)	1 (7.7%)	0.342
Hyperlipidemia Ex*, n (%)	2 (8.0%)	3 (23.1%)	0.315
Hormonal disease (thyroid, pregnancy etc.) Ex*, n(%)	1 (4.0%)	0 (0.0%)	>0.999
Hematological diseases Ex*, n(%)	1 (4.0%)	1 (7.7%)	>0.999
Cardio Vascular disease Ex* n(%)	1 (4.0%)	3 (23.1%)	0.107
Neurological event Ex*, n(%)	1 (4.0%)	1 (7.7%)	>0.999
Other Retinopathic disease DM, HTN Dx, n(%)	0 (0.0%)	0 (0.0%)	NA
Other Retinopathic disease DM HTN Ex, n (%)	1 (4.0%)	0 (0.0%)	>0.999
Local steroids treatment Ex* n (%)	8 (32.0%)	4 (30.8%)	>0.999
Local glaucoma, (number of medications) treatment Ex* n(%)			
0	23 (92.0%)	13 (100.0%)	>0.999
2	1 (4.0%)	0 (0.0%)	
3	1 (4.0%)	0 (0.0%)	
Local NSAID treatment Ex*, n(%)	0 (0.0%)	2 (15.4%)	0.111
Active anterior uveitis Dx** 0–4, n (%)			
0	7 (28.0%)	9 (69.2%)	**0.023**
1	15 (60.0%)	2 (15.4%)	
2	2 (8.0%)	2 (15.4%)	
3	1 (4.0%)	0 (0.0%)	
Active anterior uveitis Ex* 0–4, n(%)			
0	22 (88.0%)	10 (76.9%)	0.648
0.5	0 (0.0%)	1 (7.7%)	
1.0	2 (8.0%)	1 (7.7%)	
1.5	1 (4.0%)	1 (7.7%)	
Keratic Precipitate Dx**, n(%)	11 (44.0%)	3 (23.1%)	0.294
Keratic Precipitate Ex*,	2 (8.0%)	3 (23.1%)	0.315
Posterior synechia Dx** 0–4, n(%)			
0	20 (80.0%)	12 (92.3%)	0.245
1	2 (8.0%)	0 (0.0%)	
2	3 (12.0%)	0 (0.0%)	
4	0 (0.0%)	1 (7.7%)	
Posterior synechia Ex* 0–4, n(%)			
0	23 (92.0%)	12 (92.3%)	0.414
1	1 (4.0%)	0 (0.0%)	
2	2 (8.0%)	1 (7.7%)	
Iris nodules Dx**, n (%)	0 (0.0%)	0 (0.0%)	NA
Iris nodules Ex*, n (%)	1 (4.0%)	0 (0.0%)	>0.999
Lens Dx** Cataract nuclear sclerosis, n (%)			
0	23 (92.0%)	12 (92.3%)	0.428
1	2 (8.0%)	0 (0.0%)	
2	0 (0.0%)	1 (7.7%)	
Lens Ex*Cataract nuclear sclerosis, n (%)	6 (24.0%)	1 (7.7%)	0.385
Lens Dx** Cataract Posterior sub capsular, n (%)			
0	16 (64.0%)	1 (7.7%)	0.132
1	9 (36.0%)	10 (76.9%)	
2	0 (0.0%)	2 (15.4%)	
Lens Ex* Cataract Posterior sub capsular, n (%)			
0	18 (72.0%)	11 (84.6%)	0.139
1	7 (28.0%)	1 (7.7%)	
2	0 (0.0%)	1 (7.7%)	
Pseudophakia Dx**, n (%)	1 (4.0%)	0 (0.0%)	>0.999
Pseudophakia Ex*n (%)	6 (24.0%)	1 (7.7%)	0.385
Vitritis Dx**, n (%)	24 (96.0%)	13 (100.0%)	>0.999
Vitritis Ex*, n (%)	18 (72.0%)	6 (46.2%)	0.163
Pars planitis Dx** 0–4 clinical severity asseamnet, n (%)			
0	3 (12.0%)	0 (0.0%)	0.229
1	15 (60.0%)	12 (92.3%)	
2	6 (24.0%)	1 (7.7%)	
3	1 (4.0%)	0 (0.0%)	
Pars planitis Ex* 0–4 clinical severity asseamnet, n (%)			
0	9 (36.0%)	7 (53.8%)	0.494
1	11 (44.0%)	6 (46.2%)	
2	3 (12.0%)	0 (0.0%)	
3	2 (8.0%)	0 (0.0%)	
Vitreal cells Dx**, 0–4, n (%)			
0	3 (12.0%)	2 (15.4%)	0.149
1	10 (40.0%)	7 (53.8%)	
2	10 (40.0%)	1 (7.7%)	
3	2 (8.0%)	2 (15.4%)	
4	0 (0.0%)	1 (7.7%)	
Vitreal cells Ex*, 0–4, n (%)			
0	10 (40.0%)	7 (53.8%)	0.835
1	2 (8.0%)	0 (0.0%)	
2	11 (44.0%)	5 (38.5%)	
Vitreal snow balls Dx**, 0–4 quadrants, n (%)			
0	5 (20.0%)	4 (30.8%)	**0.035**
1	4 (16.0%)	6 (46.2%)	
2	15 (60.0%)	2 (15.4%)	
4	1 (4.0%)	1 (7.7%)	
Vitreal snow balls Ex*, 0–4 quadrants, n (%)			
0	10 (40.0%)	4 (30.8%)	0.749
1	8 (32.0%)	7 (53.8%)	
2	4 (16.0%)	2 (15.4%)	
3	2 (8.0%)	0 (0.0%)	
4	1 (4.0%)	0 (0.0%)	
Vitreal haze, Dx** 0–4 SUN classification, n (%)			
0	2 (8.0%)	4 (30.8%)	0.063
1	11 (48.0%)	3 (23.1%)	
2	10 (40.0%)	3 (23.1%)	
3	1 (4.0%)	3 (23.1%)	
Vitreal haze Ex*, 0–4 SUN classification, n (%)			
0	10 (40.0%)	5 (38.5%)	0.219
1	13 (52.0%)	4 (30.8%)	
2	2 (8.0%)	4 (30.8%)	
Vitreal snow banck Dx**, 0–4 quadrants, n (%)			
0	20 (80.0%)	9 (69.2%)	0.607
1	4 (16.0%)	4 (30.8%)	
4	1 (4.0%)	0 (0.0%)	
Vitreal snow banck Ex*, 0–4 quadrants, n (%)			
0	23 (92.0%)	9 (69.2%)	**0.037**
1	1 (4.0%)	4 (30.8%)	
2	1 (4.0%)	0 (0.0%)	
Vitreal strands Dx**, 0–4 quadrants, n (%)			
0	22 (88.0%)	9 (69.2%)	0.228
1	1 (4.0%)	1 (7.7%)	
2	1 (4.0%)	2 (15.4%)	
3	0 (0.0%)	1 (7.7%)	
4	1 (4.0%)	0 (0.0%)	
Vitreal strands Ex*, 0–4 quadrants, n (%)			
0	9 (36.0%)	8 (61.5%)	0.361
1	12 (48.0%)	4 (30.8%)	
2	4 (16.0%)	1 (7.7%)	
Retinitis Dx** 0–4, n(%)	0 (0.0%)	0 (0.0%)	NA
Retinitis Ex* 0–4, n(%)	0 (0.0%)	0 (0.0%)	NA
Optic disk abnormality (pale, edema, NVD, optic neuritis etc.) Dx**, n (%)	1 (4.0%)	0 (0.0%)	>0.999
Optic disk abnormality (pale, edema, NVD, optic neuritis etc.) Ex*, n (%)	3 (12.0%)	0 (0.0%)	0.538
CME Dx**, n(%)	5 (20.0%)	2 (15.4%)	>0.999
CME Ex*, n(%)	1 (4.0%)	1 (7.7%)	>0.999
ERM Dx**, n(%)	1 (4.0%)	1 (7.7%)	>0.999
ERM Ex*, n(%)	5 (20.0%)	2 (15.4%)	>0.999
Active vasculitis at Dx**, 0–4 quadrants, n (%)			
0	18 (72.0%)	11 (84.6%)	0.853
1	4 (16.0%)	1 (7.7%)	
2	3 (12.0%)	1 (7.7%)	
Non active vasculitis at Dx**, 0–4 quadrants, n (%)			
0	25 (100.0%)	12 (92.3%)	0.324
1	0 (0.0%)	1 (7.7%)	
Active vasculitis at Ex*, 0–4 quadrants, n (%)			
0	21 (84.0%)	11 (84.6%)	0.877
1	2 (8.0%)	1 (7.7%)	
2	0 (0.0%)	1 (7.7%)	
3	1 (4.0%)	0 (0.0%)	
4	1 (4.0%)	0 (0.0%)	
Non active vasculitis at Ex*, 0–4 quadrants, n (%)			
0	20 (80.0%)	10 (76.9%)	0.168
1	3 (12.0%)	0 (0.0%)	
2	0 (0.0%)	2 (15.4%)	
3	1 (4.0%)	0 (0.0%)	
4	1 (4.0%)	1 (7.7%)	
Other retinal abnormality (NVE, gliosis, RD etc.) Dx**, n(%)			
0	24 (96.0%)	13 (100.0%)	>0.999
1	1 (4.0%)	0 (0.0%)	
Other retinal abnormality (NVE, gliosis, RD etc.) Ex*, n(%)			
0	22 (88.0%)	11 (92.3%)	0.211
1	3 (12.0%)	0 (0.0%)	
4	0 (0.0%)	1 (7.7%)	

UV = Uveitis, MS = Multiple Sclerosis, * Ex = Examination at study antrance, iqr = interquartile range, DM = Diabetes Mellitus ** Dx = at diagnosis, CME = Cystoid Macular Edema ERM = Epiretinal Membrane, RD = Retinal Detachment, NVE = neovascularization Dx** = examination at the time of diagnosis

The multivariate analysis revealed that age and sex were not associated with EDSS 3 (age: hazard ratio [HR] 1.04, 95%confidence interval [CI] 0.97–1.11, *P* = 0.318, female: HR 1.55, 95%CI 0.55–4.31, *P* = 0.406). There was a trend for presentation with uveitis prior to MS to be associated with a lower incidence of EDSS 3 (HR 0.15, 95%CI 0.02–1.19, *P* = 0.072). The findings were the same when adjusted to the propensity score (HR 0.15, 95%CI 0.02–1.18, *P* = 0.071). The bootstrapping method demonstrated that age and sex remained unassociated with EDSS 3 (*P* = 0.307 and *P* = 0.395, respectively), but presentation with uveitis prior to MS diagnosis was significantly associated with a lower incidence of EDSS 3 (HR 0.15, 95%CI 0.001–0.59, *P* = 0.029). The findings were the same when adjusted to the propensity score (HR 0.15, 95%CI 0.001–0.58, *P* = 0.027).

## Discussion

While the existence of an association between uveitis and MS is well-recognized, the implication of that association has not been clarified. We speculated that the connection might be disease progression and conducted a long term follow-up historical cohort study in order to compare the progression of MS in patients who had been diagnosed with uveitis prior to their MS diagnosis with the progression of MS in those without uveitis. All of the former patients had presented with intermediate uveitis along with a range of signs, including anterior uveitis, posterior uveitis, and vasculitis. These findings correlate with the reports in the literature that the most common type of uveitis in MS is intermediate uveitis that primarily involves the vitreous, peripheral retina, and pars plana [8, 10, 11]. The ocular inflammation may develop concurrently, prior to, or after the development of the neurological signs and symptoms, but none of the earlier studies had identified any major difference in the clinical features of MS patients with and without uveitis [12].

Our current results demonstrate that the patients in the study group had a better neurological prognosis than the control group. The univariate analysis failed to demonstrate a significant association but rather only a trend, while a bootstrapping analysis which randomly samples 1000 times the number of patients with replacement revealed a significant association. Similarly Shugaiv et al reported that uveitis might be used as a good prognostic factor for MS patients [7]. Their retrospective report compared 41 MS patients with uveitis and 100 randomly selected MS patients with no uveitis in a cross sectional study: none of the uveitis patients had developed progressive forms of MS during their follow-up, whereas 36% of the MS patients without uveitis had primary or secondary progressive MS (*P* < 0.001). Moreover, the final EDSS and progression index (PI) scores of the MS patients with uveitis were significantly lower than those without uveitis (*P* = 0.004 and *P* < 0.001, respectively). Our current long term follow up historical cohort study sought to determine the association between the presence of a uveitis diagnosis prior to an MS diagnosis and no pre-existing uveitis diagnosis in MS patients. We found as hypothesized that presentation with uveitis prior to neurological symptoms of MS involved statistically significant better disease progression.

We hypothesize that one possible explanation for the differences between the study groups could be the early identification of an autoimmune illness (in the form of uveitis) in our cohort that may have led to early immunomodulatory treatment that may, in turn, have altered the form and severity of the MS in these patients. It would take a larger multicenter database to evaluate ours and Shugaiv et al’s [7] findings. Moreover, Forooghian et al found strong evidence in the literature which demonstrated that uveitis in MS may, in fact, be a component of the MS disease process [13]. They stated that the retina is a non-myelinated component of the CNS, but the processes that occur in the retina in MS-associated uveitis are similar to those that occur in other myelinated parts of the CNS of MS patients. Thus, the fact that the retina lacks myelin should not exclude it as a potential site for MS disease involvement [13].

Based on our findings and those of others [14], it appears that there is sufficient evidence to conclude that uveitis in MS patients is, in fact, a component of the MS disease process related to the immunological pathology that occurs in MS, rather than an isolated and unrelated comorbid autoimmune disease that occurs in MS patients. Taken together, these lines of evidence support our hypothesis that early identification of an autoimmune illness and early immunomodulatory treatment (in this case, for uveitis) may have altered the form and severity of the MS in patients with coexisting uveitis see Table 1.

One major limitation of this study is the small sample size. Another is the lack of ophthalmic examinations of patients who did not report symptoms of uveitis at the time of MS diagnosis, possibly resulting in a classification bias which may reduce the statistical significance of our results.

In conclusion, the MS patients who presented with uveitis that preceded their neurological symptoms demonstrated better neurological prognosis (EDSS) and thereby point to the association between a uveitis diagnosis prior to an MS diagnosis and MS progression. It is essential to establish a larger multicenter database for further studies to evaluate our findings.

## Abbreviations

EDSS: Expanded Disability Status Scale
MRI: Magnetic Resonance Imaging
MS: Multiple Sclerosis
PI: Progression Index
PPMS: Primary Progressive MS
RRMS: Relapsing–Remitting MS
SUN: Standardization of Uveitis Nomenclature
